# Keratocystic Odontogenic Tumor with an Ectopic Tooth in Maxilla

**DOI:** 10.1155/2013/232096

**Published:** 2013-12-11

**Authors:** Basavaraj T. Bhagawati, Manish Gupta, Gaurav Narang, Sharanamma Bhagawati

**Affiliations:** ^1^Department of Oral Medicine and Radiology, Shree Bankey Bihari Dental College, Masuri, Ghaziabad, Utta Pradesh-201302, India; ^2^Department of Periodontology, Shree Bankey Bihari Dental College, NH-24, Masuri, Ghaziabad, Utta Pradesh-201302, India

## Abstract

The term odontogenic keratocyst was first used by Philipsen in the year 1956. The lesion was renamed by him as keratocystic odontogenic tumor (KCOT) and reclassified as odontogenic neoplasm in the World Health Organization's 2005 edition that occurs commonly in the jaws having a predilection for the angle and ascending ramus of mandible. In contrast, KCOTs arising in the maxillary premolar region are relatively rare. Here, we discuss a rare case of keratocystic odontogenic tumor occurring in the maxilla with an ectopic tooth position.

## 1. Introduction

Keratocystic odontogenic tumor (KCOT) is defined as “a benign uni- or multicystic, intraosseous tumor of odontogenic origin, with a characteristic lining of parakeratinized stratified squamous epithelium and potential for aggressive, infiltrative behavior.”

In 2005, the World Health Organization redefined the odontogenic keratocyst as a result of its biological behavior as a benign tumor of odontogenic origin and named it as keratocystic odontogenic tumor.

KCOTs comprise approximately 11% of all cysts of the jaws. They occur most commonly in the mandible, especially in the posterior body and ramus regions. They almost always occur within bone, although a small number of cases of peripheral KCOT have been reported.

## 2. Case Report

**Figure 1:**
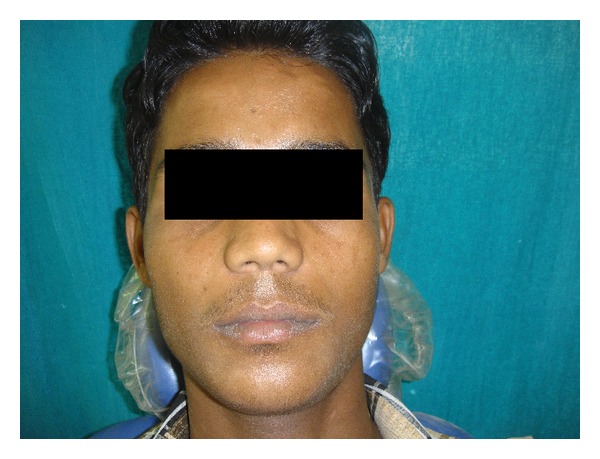
Profile photograph of the patient.

A 17-year-old male patient came to the department with a chief complain of pus discharge from the right upper back teeth region 3-4 months ago with pain and swelling 15−20 days ago. Pain was gradual in onset, throbbing type, continuous, nonradiating, aggravates on mastication, and relieved on taking medication. Swelling was initially smaller in size and gradually increased to the present size associated with pus discharge from right upper back region 2-3 months ago. There was no history of trauma or fever. Patient also gave history of exfoliation of a tooth from right upper back tooth region on its own (Figure 1).

On general physical examination, patient was found to be moderately built and nourished. He was conscious, cooperative, and well oriented with time, place, and person. Extraoral examination showed no abnormality.

On intraoral examination, a localized, solitary swelling was present in the right upper back vestibular region measuring approximately 1–1.5 cm in diameter in relation to teeth 15, 16 with overlying mucosa slightly erythematous in appearance; however the surface appeared to be smooth and the surrounding area appeared normal. On palpations all inspectory findings were confirmed. The swelling was nontender, bony hard in consistency, nonfluctuant, nonpulsatile, noncompressible, and nonreducible. Hard tissue examination revealed missing 13, 14 and over retained 53. On percussions 15, 16 were tender. All other teeth were present in the oral cavity with no abnormality. On the basis of history and clinical examination, a provisional diagnosis of dentigerous cyst was given.

**Figure 2:**
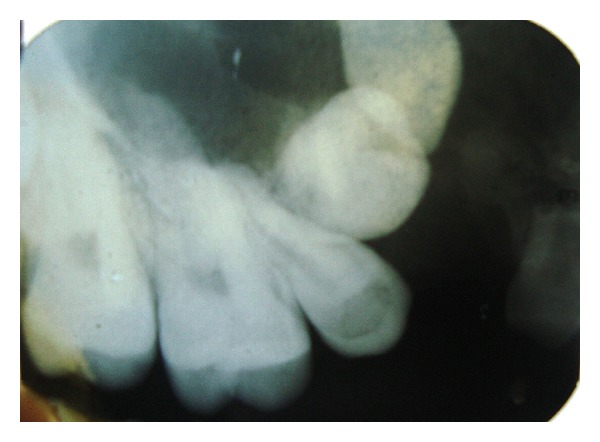
IOPA showing diffuse radiolucency in the region 15, 16, overretained 53 and crowns of 13, 14.

**Figure 3:**
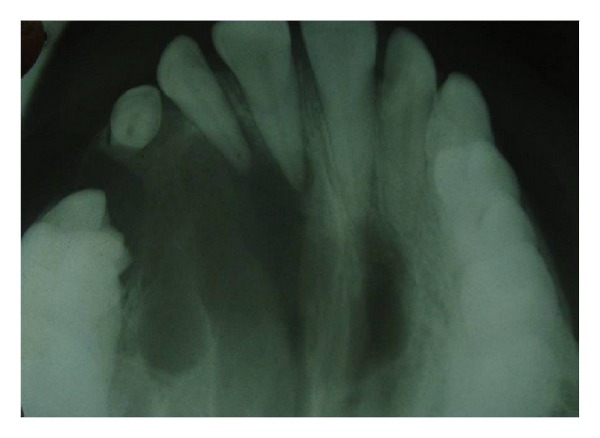
Maxillary cross-sectional occlusal radiograph showing radiolucency involving the right maxilla.

**Figure 4:**
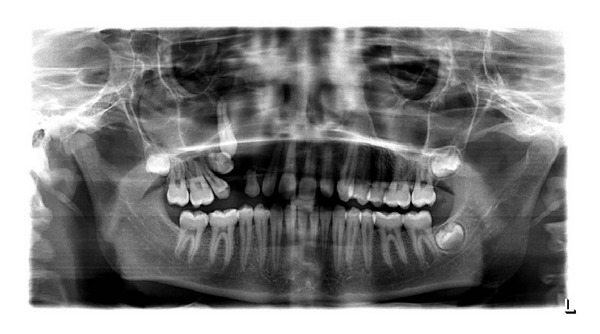
Panoramic radiograph showing impacted 13, 14.

Patient was further subjected to radiographic investigation where intraoral periapical radiograph (Figure 2), occlusal maxillary cross-sectional (Figure 3), and panoramic radiograph (Figure 4) were done. IOPA revealed an ill-defined radiolucency around the apices of 15, 16 with poorly defined borders. Occlusal radiograph showed ill-defined radiolucency with intermittent septa involving the entire right maxilla and crossing the midline.

Panoramic radiograph revealed multilocular radiolucency separated with intermittent septa involving the right maxilla with impacted canine and first premolar. Displacement of second premolar is also seen.

**Figure 5:**
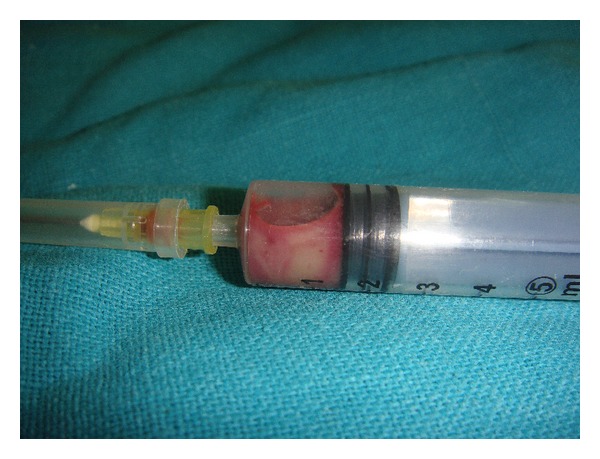
Fine needle aspiration cytology showing straw colored fluid intermixed with blood.

Fine needle aspiration of the cyst was done which revealed straw colored fluid intermixed with blood (Figure 5). The sample was sent for protein estimation which revealed 3.2 gm/dL protein content.

**Figure 6:**
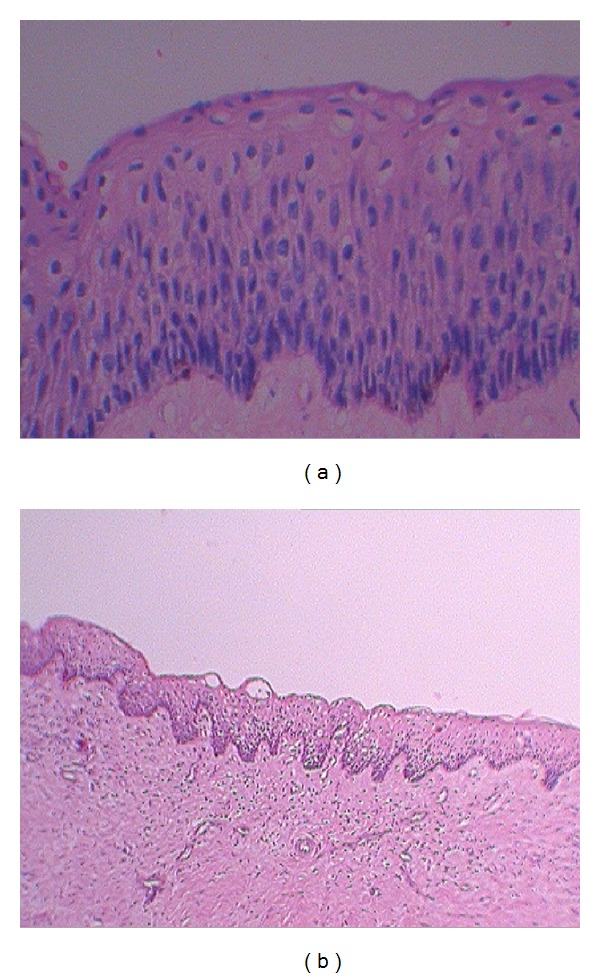
Photomicrograph of the histopathological slide.

Surgical excision of the lesion was done and was sent for histopathological confirmation. Histopathologic features reveal cystic lining with parakeratinized, corrugated stratified squamous epithelium exhibiting palisading basal cells, nuclear hyperchromatism, and flattened epithelial connective tissue junction at places. The underlying connective tissue capsule is fibrillar with admixed population of acute and chronic inflammatory cells at places, blood vessels, and peripheral bone. Features are suggestive of keratocystic odontogenic tumor (Figures 6(a) and 6(b)).

## 3. Discussion

Keratocystic odontogenic tumor (KCOT), formerly known as OKC, is a benign unicystic or multicystic intraosseous neoplasm of odontogenic origin which arises from the remnants of the dental lamina both in mandible and maxilla [1].

The discovery of increased mitotic activity in the cyst epithelium, the potential for epithelial budding from basal layer or daughter cysts in the cyst wall, the presence of chromosomal abnormalities, and the role of mutation of the PTCH gene in the etiology of KCOT resulted in its reclassification and renaming as keratocystic odontogenic tumor [1–6].

Keratocystic odontogenic tumors are sometimes related to nevoid basal cell carcinoma syndrome, which is a rare inheritance disorder caused by mutations in the PTCH gene on chromosome 9 causing multiple odontogenic keratocyst of the jaws, basal cell carcinoma (BCC) of the skin, and vertebral anomalies [7].

According to Madras and Lapointe three factors led to the recharacterization of the keratocyst as KCOT. The KCOT exhibits locally destructive and highly recurrent behavior; the histopathology of the KCOT reveals budding of the basal layer into the connective tissue and frequent mitotic figures, and, finally, the KCOTs are associated with an inactivation of PCHT, the tumor suppressor gene [8].

KCOT has a predilection for occurring in the mandible (75.58%) as compared to maxilla [9–12]. In mandible, majority occur in third molar-ramus area, followed by first and second molar, and then followed by anterior mandible. In maxilla, the most common site is the third molar area followed by the cuspid region [9, 10, 13, 14].

KCOT when involving the maxilla sinus must be carefully assessed because the orbital damage and the spreading of associated infections could lead to local and systemic compromise to the patient, present with pain. Displacement of tooth and destruction of the floor of the orbit and proptosis of eyeball when it involves maxilla are most common features [15].

The ectopic eruption of teeth in the regions other than the oral cavity is rare, although there have been reports of teeth in unusual locations, one of them being the maxillary sinus. The etiology of ectopic eruption has not yet been completely clarified but may occur as a result of trauma, infection, developmental anomalies, and pathologic condition such as odontogenic cysts. As the growth of an odontogenic cyst continues, the cyst encroaches on the space of the sinus and displaces its borders: it may be that the displacement of teeth buds by this expansion of a cyst results in the ectopic eruption of a tooth [16].

KCOT is more common in males than females and occurs over a wide age range and is typically diagnosed during second to fourth decade [9].

On radiographic examination, KCOT cannot be distinguished from other intrabony cysts. In mandible, the epicenter is commonly located superior to the inferior alveolar nerve canal. It usually shows evidence of a cortical border with a scalloped outline which represents variation in the growth pattern of the cyst [13]. An important characteristic of KCOT is its propensity to grow along the internal aspect of jaws causing minimal expansion [9].

The keratocystic odontogenic tumor wall is usually thin unless there has been a superimposed inflammation [9, 10, 13, 14]. Characteristic features are

- a parakeratinized surface which is typically corrugated, rippled, or wrinkled;
- uniformity of thickness of epithelium ranging from 6 to 10 cells thick;
- a prominent palisaded, polarized basal cell layer of cells having “picket fence” or “tomb stone” appearance.

Numerous surgical modalities have been suggested for the treatment of KCOTs, including enucleation with primary closure, enucleation with open packing, and resection with or without loss of jaw continuity. The treatment depends on several factors, such as age, location, and size of lesion and whether the lesion is primary or recurrent. Total enucleation with or without “peripheral ostectomy” is the treatment of choice for most KCOTs unless lesion is recurrent or has significantly invaded soft tissue [9, 17].

## 4. Conclusion

The destructive, high recurrence potential of KCOTs and their ability to resemble other jaw cysts make it important to consider them in differential diagnosis of radiolucent lesions occurring in the maxilla. Also due to aggressive behavior and high recurrence rate of KCOT, all pathologic tissue should be properly excised and histopathologic confirmation should be made for a definitive diagnosis. The followup is advised for every six months for the next two years.
